# Etching-enabled extreme miniaturization of graded-index fiber-based optical coherence tomography probes

**DOI:** 10.1117/1.JBO.25.3.032006

**Published:** 2019-11-09

**Authors:** Alexandre Abid, Shiv Mittal, Christos Boutopoulos

**Affiliations:** a University of Montreal, Institute of Biomedical Engineering, Montreal, Quebec, Canada; b Maisonneuve-Rosemont Hospital Research Centre, Montreal, Quebec, Canada; c University of British Columbia, Faculty of Applied Science, Vancouver, British Columbia, Canada; d University of Montreal, Department of Ophthalmology, Montreal, Quebec, Canada

**Keywords:** optical fiber probe, miniaturized, optical coherence tomography, chemical etching, graded-index fiber

## Abstract

We introduced and validated a method to miniaturize graded-index (GRIN) fiber-based optical coherence tomography (OCT) probes down to 70  μm in diameter. The probes consist in an assembly of single-mode (SM), coreless (CL), and graded-index (GRIN) fibers. We opted for a probe design enabling controlled size reduction by hydrogen fluoride etching. The fabrication approach prevents nonuniform etching for both the GRIN and SM fiber components, while it requires no probe polishing postetching. We found that the miniaturized probes present insignificant loss of sensitivity (∼1  dB) compared to their thicker (125  μm) counterparts. We also showed that their focusing capabilities remain tunable and highly predictable. The fabrication process is simple and can be carried out by using inexpensive telecom equipment. Both the fabrication process and the developed probes can benefit the prototyping of minimally invasive endoscopic tools.

## 1 Introduction

Optical coherence tomography (OCT) is a noninvasive tomographic imaging technique based on low-coherence optical interferometry.^1^ It can achieve high spatial resolution (10’s μm down to sub-μm), similar to the one attained by histology. However, OCT is limited to few mm imaging depth in tissue. To overcome this intrinsic limitation, several research groups have developed endoscope prototypes integrating OCT imaging capabilities. These devices have found applications in the ophthalmic, cardiovascular, and gastrointestinal tract systems.^2^^,^^3^ Common designs include miniaturized fiber-based OCT probes encased within medical devices such as capsules and needles.^2^^,^^4^ The performance of these systems is largely based on the ability to optimize light delivery to a tissue of interest, as well as backscattered light collection from it. Sensitivity and resolution optimization can be attained by integrating an application-tailored microlens at the distal end of an OCT fiber probe.^4^^–^^9^

**Fig. 1:**
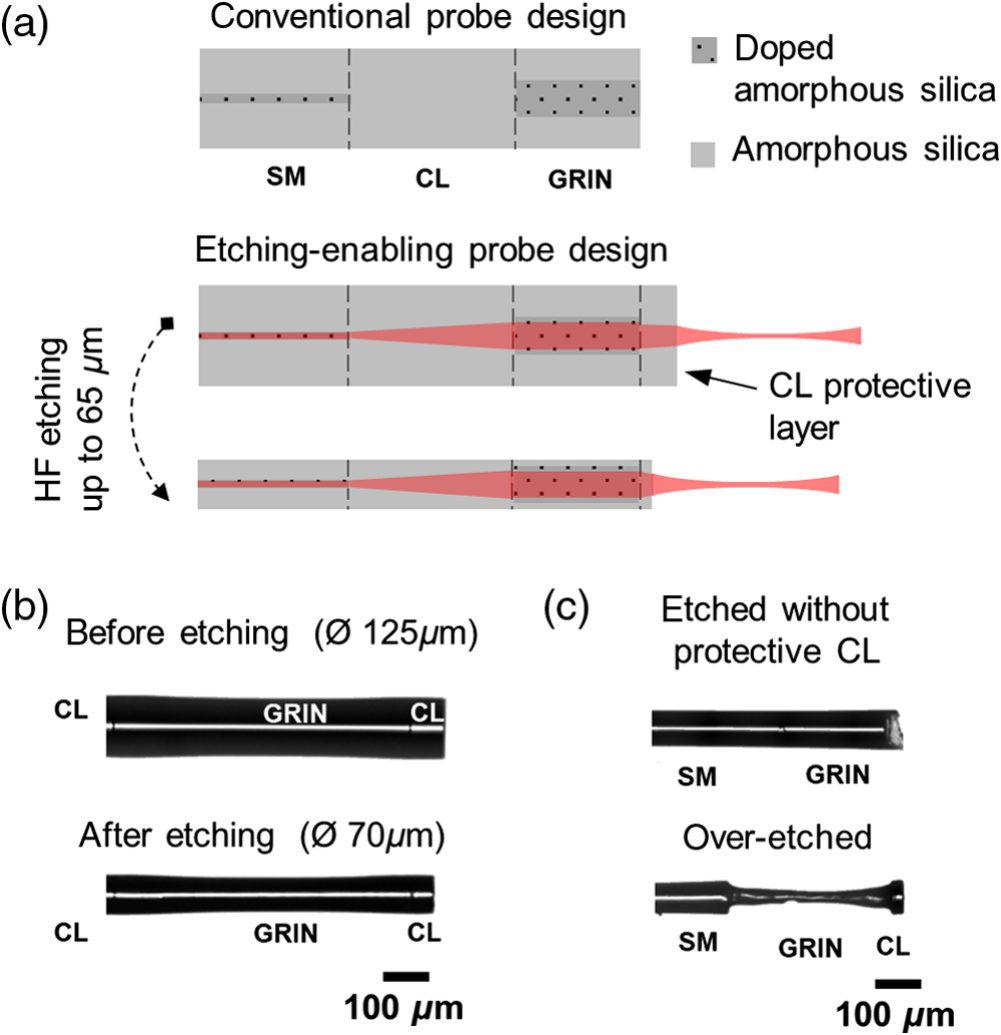
(a) Schematic showing conventional and etching-enabling designs of forward imaging OCT probes. (b) Microscopy images of an OCT probe prior and after controlled etching. (c) Indicative microscopy images of noncontrolled etched probes because of the GRIN core exposure to HF. SM stands for single-mode fiber; CL, core-less fiber; and GRIN, graded-index fiber.

A commonly used microlens design consists of an assembly of a single-mode (SM) fiber fusion-spliced to a coreless (CL) and a graded-index (GRIN) fiber^6^^,^^9^ [Fig. 1(a)]. Such GRIN-based probe design can be implemented by using commercial optical fiber components and affordable fabrication tools. It enables fabrication of probes with controlled optical properties in both forward and side viewing imaging configurations.^5^^,^^6^ Even though 80-μm SM fibers are today commercially available, the smallest commercially available GRIN and CL fibers remain 125  μm in diameter. This imposes a 125-μm miniaturization limit for focusing GRIN-based OCT probes fabricated with a splicing approach.

Alternative non-GRIN-based fabrication approaches have enabled probe miniaturization below 125  μm. Marrese et al. recently fabricated a 70-μm probe based on an assembly of an etched SM fiber and a barium titanate microsphere.^8^ By integrating a high refractive index ball lens, this probe can enable optimized performance in common path configurations. However, the fabrication process is challenging since precise positioning and fixing of a microsphere to the tip of the etched SM fiber is required. Lee et al.^10^ used chemical etching to miniaturize lensless side viewing probes, composed of three different fiber components of progressively increased core diameter. These probes significantly extend the effective imaging depth compared to a SM fiber. However, this GRIN-less, nonfocusing design results to compromised lateral resolution compared to lens-based probes, while the miniaturization approach requires a challenging polishing step postetching.

Given the versatility and wide use of GRIN-based probes, the development of a simple fabrication approach facilitating extreme miniaturization without compromising the simplicity and tunability of a GRIN-based design can benefit a variety of OCT applications. For example, it can enable integration with minimally invasive medical needles. Note that although 41G needles (inner diameter: 90  μm) are today widely available, existing GRIN-based probes can only be encased within 30G (inner diameter: 159  μm) or thicker needles.

In this work, we present a simple method enabling controllable miniaturization of focusing GRIN-based OCT probes down to 70  μm in diameter. This represents ∼45% size reduction compared to previously reported focusing ultrathin probes based on commercial GRIN fibers.^6^^,^^9^ Size reduction was achieved without introducing complicated fabrication steps and/or using special equipment. With beam profiling and OCT measurements, we show that the fabricated miniaturized probes possess tunable optical properties, uncompromised compared to their thicker counterparts.

## 2 Experimental

We used commercially available fibers (SM: SM800-5.6-125, CL: FG125LA, GRIN: GIF625, Thorlabs) and inexpensive telecom splicing/cleaving equipment (AI-6, Signal Fire, China) for this work.

We etched the probes by immersing them in a plastic [resistant to hydrogen fluoride (HF)] container filled with 48% HF solution (HX0621, Millipore) for 15 to 18 min at room temperature. The length of the etched part of each fiber probe was 2 cm.

For the beam profiling measurements, we coupled an infrared laser (central wavelength: 840, bandwidth: 50 nm, SLD-37-HP, Superlum, Ireland) to the probes. We used a microbeam profiler setup, composed of a 32× objective lens (NA=0.4), an achromatic lens (AC254-150-A, Thorlabs), and a CCD camera (MV-GE200GM, Mindvision Technology) to acquire cross-section beam profiles in air. We employed the same setup to visualize the exiting beam profile in liquid. Exiting beam profiles in liquid were acquired perpendicular to the propagation axis. For these experiments, we immersed the probes in a cuvette filled with a water-soluble infrared dye (IR-806, Sigma-Aldrich). To attain the desired field of view, we either used a 5× (NA=0.12) or a 10× (NA=0.25) objective lens.

OCT measurement was carried out using a common path configuration. We used a homemade spectral-domain OCT (SD-OCT) setup, composed of the laser source described above, an optical isolator (850-nm multimode isolator, AC Photonics Inc.), a 90:10 fiber coupler (TW850R2A2, Thorlabs), and a compact spectrometer (USB 4000, Ocean Optics). The spectrometer was equipped with an 1800  lines/mm grating (Grating #11, Ocean Optics) and a linear 3648-element (8  μm×200  μm) CCD array (Toshiba TCD1304AP). Its optical resolution was 0.16 nm for the used fiber. We used a LabVIEW-based program to apply spectral reshaping and control the system. The sensitivity was determined to be 85 dB in a common path configuration using a nonfocusing probe. A-scans were acquired by setting the exposure time to 1000  μs and the output power at the probe exit to 270  μW. SNR (dB) was calculated as $20\times \log(I_{s-b}/\sigma_{b})$, where $I_{s-b}$ is the background-subtracted signal and $\sigma_{b}$ is the standard deviation of the background signal. Background signal was acquired with no sample present.

We used freshly enucleated porcine eyes to acquire comparative retinal B-scans. Prior to imaging, we placed the porcine eyes on a holder and removed the cornea and iris by dissecting a circular segment of the sclera. We also removed the lens but not the vitreous. The probes were immersed into the vitreous during the measurement. For both porcine eye and scotch tape imaging, we mounted the probes on a metallic post and used a motorized translation stage to scan the samples with a 1-μm lateral scanning step. We did not apply signal averaging.

## 3 Results and Discussion

### 3.1 Probe Miniaturization

**Fig. 2:**
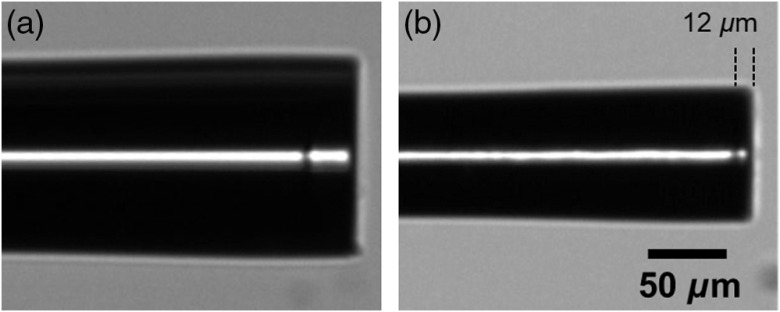
Optical microscopy images of an OCT probe (a) before and (b) after etching.

To achieve extreme miniaturization, we sought for a probe design allowing controllable size reduction with HF wet etching. We opted for a widely used probe configuration, an assembly of SM, CL, and GRIN fibers,^5^ with an addition of a short CL fiber component at the distal end [Fig. 1(a)]. This key modification is crucial for controlling the postetching morphology and performance of the probes because it protects the GRIN fiber core from nonuniform etching. Prior miniaturization, the probes were 125  μm in diameter [Fig. 1(b)], composed of a doped amorphous silica core (5.6  μm for SM and 62.5  μm for GRIN), and a nondoped amorphous silica cladding [Fig. 1(a)]. Note that HF is capable of etching nondoped amorphous silica isotropically but etches doped amorphous silica nonuniformly and with much faster rate. Since the cladding of the SM and GRIN fibers and the entire CL fiber are made of nondoped amorphous silica, controlled size reduction of the probe diameter can be achieved by tuning the etching time [Fig. 1(b)]. Note that we observed no deterioration of the protective CL layer for etching down to 12  μm in thickness (Fig. 2). To illustrate the importance of the CL protective layer, we also etched probes that had no such layer (i.e., conventional design). Contrary to the etching-enabling approach, the GRIN fiber core is exposed to HF. As a result, the morphology of the etched probe’s tip becomes nonuniform and hardly predictable [Fig. 1(c), top probe]. Note that the diameter of the GRIN fiber core determines the miniaturization limit, which is equal to 62.5  μm for the GIF625 fiber used in this work. Figure 1(c) (bottom probe) illustrates the dramatic effect of HF overetching on the integrity of the GRIN fiber component (i.e., core deterioration). These results demonstrate that the morphology of the miniaturized probes remains unaffected compared to their thicker counterparts if (i) an etching-enabling design is employed and (ii) the miniaturization limit, imposed by the GRIN core diameter, is respected.

Note that HF etching has been shown to increase the surface roughness of the etched fiber.^11^ This effect has been attributed to the deposition of the etching products on the surface of the fiber. For long etching time, especially when the core is reached, micrometric scale defects on the fiber surface can be formed.^11^ With overetched probes being an exception [Fig. 1(c)], we did not observe such defects for the etching conditions used in this study. However, in some occasions, we observed isolated defects around the splicing points. The miniaturization process has been found to be highly reproducible as the etching rate variability was negligible. Although the miniaturized probes presented increased suppleness compared to their thicker counterparts, no special handling equipment was necessary to perform the measurements presented in this article.

### 3.2 Probe Design and Optical Validation

We routinely fabricated miniaturized probes by varying the CL and GRIN fiber length, to evaluate experimentally whether the miniaturization process affects their lensing performance. We used previously published analytic expressions for the working distance (WD) and beam waist (BW) of the probes^12^ as guide for the selection of the GRIN and CL fiber lengths. The MATLAB code used for the calculations can be found freely online and executed through Code Ocean.^13^ We designed three different probes, a slightly diverging probe (probe 1; before etching: ${\mathrm{CL}}_{\mathrm{be}1}=0\;\mu m$, ${\mathrm{GRIN}}_{\mathrm{be}1}=271\;\mu m$, protective ${\mathrm{CLp}}_{\mathrm{be}1}=77\;\mu m$, ${\mathrm{OD}}_{\mathrm{be}1}=125\;\mu m$), a short WD probe (probe 2; before etching: ${\mathrm{CL}}_{\mathrm{be}2}=272\;\mu m$, ${\mathrm{GRIN}}_{\mathrm{be}2}=706\;\mu m$, protective ${\mathrm{CLp}}_{\mathrm{be}2}=112\;\mu m$, ${\mathrm{OD}}_{\mathrm{be}2}=125\;\mu m$), and long WD probe (probe 3; before etching: ${\mathrm{CL}}_{\mathrm{be}3}=272\;\mu m$, ${\mathrm{GRIN}}_{\mathrm{be}3}=678\;\mu m$, protective ${\mathrm{CLp}}_{\mathrm{be}3}=88\;\mu m$, ${\mathrm{OD}}_{\mathrm{be}3}=125\;\mu m$).

We etched the probes following the protocol given at the experimental part. The etching process affected only the diameter of the probes and the length of protective CL, while the splicing points remained intact [Figs. 1(b) and 2]. We determined the axial etching rate to be 1.406  μm/min for all fiber components forming the probe, while we calculated an etching rate of 1.875  μm/min for the longitudinal axis of the protective CL.

**Fig. 3:**
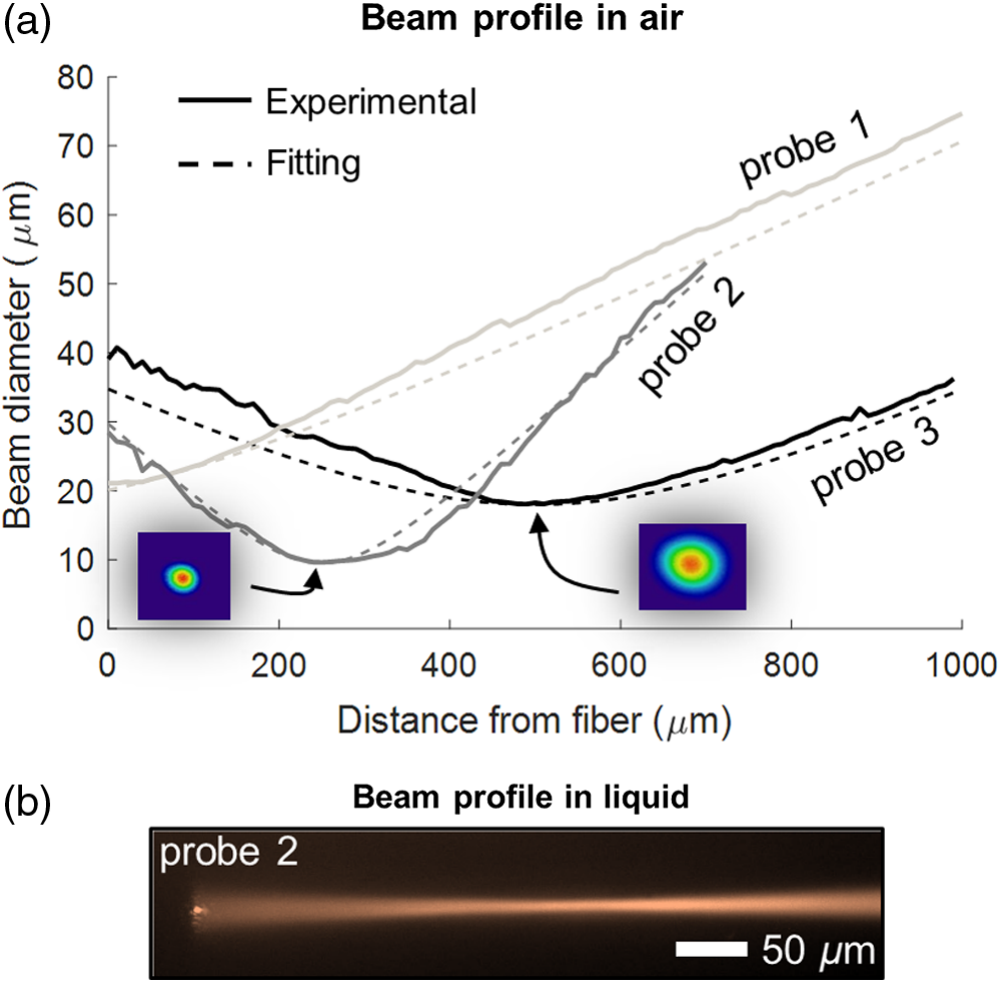
Experimental exiting beam profiles for three different ultrathin probes in air. (a) The two insets show experimental beam intensity profiles at focus. (b) An indicative exiting beam profile acquired by immersing probe 2 in an infrared fluorescent dye solution.

Figure 3 shows the beam profiling measurements for the miniaturized probes and Table 1 summarizes their predicted and experimentally measured optical properties. With paraxial Gaussian beam fitting, we measured the experimental WD in air for probe 2 and probe 3 at 250 and 500  μm, respectively. These values are in good agreement with the theoretical ones, namely 230  μm for probe 2 and 449  μm for probe 3. We observed slightly astigmatic exiting beams for both probes [see insets in [Fig. 3(a)]. We also observed this effect in nonetched probes due to imperfect splicing points. We obtained consistent measurements with side-viewing beam profiling in liquid [Fig. 3(b)]. The WD for probe 2 and probe 3 was measured to be 304 and 605  μm, respectively. The corresponding theoretical values were 306  μm for probe 2 and 597  μm for probe 3. The theoretical and experimentally validated BW and depth of field (DF) values can also be found in Table 1, where a relatively good agreement can be appreciated. Note that the differences between predicted and experimental values lie within the usual range observed for nonetched probes. We consider the imperfect splicing process as the main cause for inaccuracies. In fact, we observed slight thickening of the probes around the splicing points.

**Table 1:** Theoretical and experimental properties of miniaturized probes.

Probe	Length	Diameter	WD (μm) in air/liquid		BW (μm) in air		DF (μm) in air	
#	$\mathrm{CL}/\mathrm{GRIN}/{\mathrm{CL}}_{p}$ (μm)	OD (μm)	Theory	Exp.	Theory	Exp.	Theory	Exp.
1	0/271/49	88	—	—	—	—	—	—
2	272/706/82	70	230/306	250/304	8.2	9.6	126	210
3	272/678/50	88	449/597	500/605	13.7	18	353	460

These results indicate that the miniaturization process does not alter the optical performance of the probes. In fact, the focusing ability of the probes remains predictable using analytic expressions.^9^^,^^12^ Although cladding etching does not change probes’ optical performance, the shortening of the protective CL shifts their WD. Therefore, the anticipated longitudinal etching rate must be considered during the designing step to attain the desired final WD.

### 3.3 Comparative OCT Performance

**Fig. 4:**
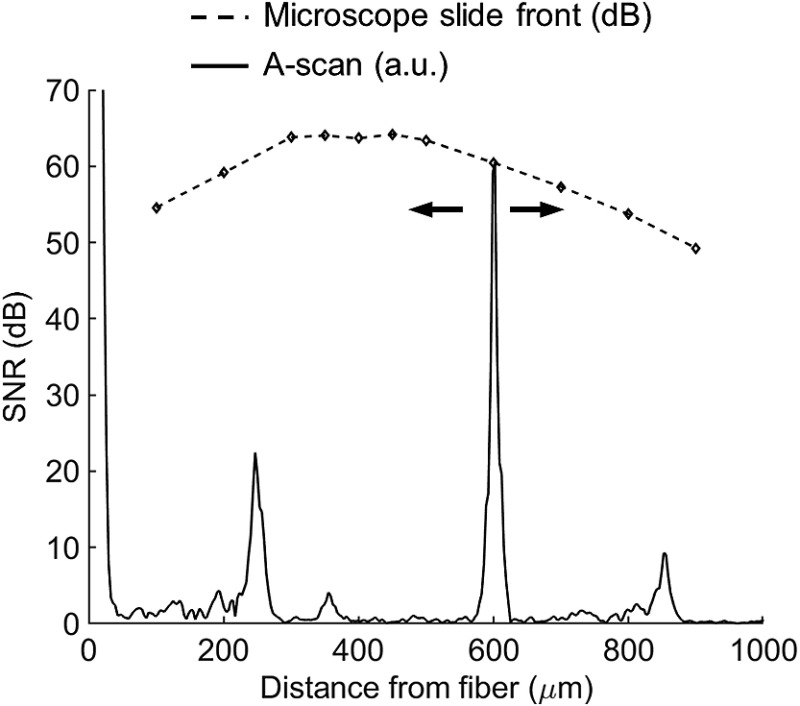
Representative A-scan (solid line) and SNR measurements (dashed line) acquired with an ultrathin focusing probe by varying its distance in respect to a glass microscope slide. The peak around 250 μm has been identified as an artifact of our setup as it was also present without connecting the probes.

**Fig. 5:**
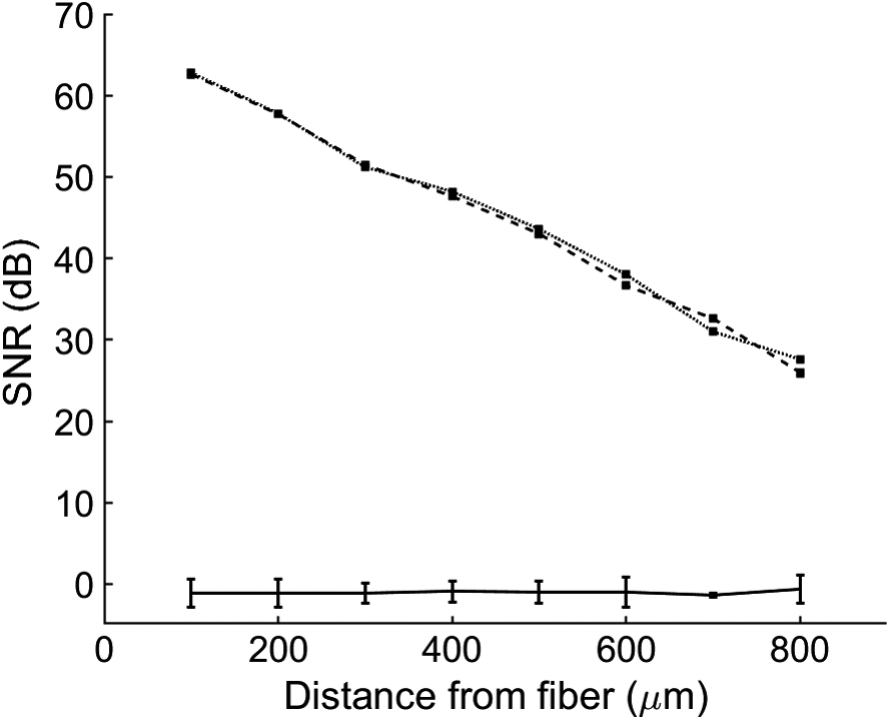
Comparison of performance for an individual probe prior (dotted line) and after etching (dashed line). The averaged difference in dB for three individual probes is also shown (solid line). The error bar indicates the standard deviation. Measurements were acquired by varying the probe distance in respect to a glass microscope slide. SNR measurements correspond to its front side (i.e., air/glass interface).

We further investigated the optical performance of the miniaturized probes by performing comparative OCT measurements. Figure 4 shows a typical A-scan of a microscope slide placed at a distance of 600  μm in front of miniaturized probe 3. We acquired SNR measurements by varying the distance between the probe and the microscope slide from 100 to 900  μm. We observed a maximum SNR for 400  μm (∼64  dB), which is consistent with the beam profile measurements obtained with the same probe [Fig. 3(a)]. To evaluate whether the miniaturization process affects the sensitivity of a given probe, we compared SNR measurements prior and after etching. Note that the etching step slightly shifts the WD of a focusing probe at a distance proportional to the protective CL shortening. To avoid confusion with the interrogated effect (i.e., splicing point and/or fiber deterioration), we used probes with slightly diverging output beams for these experiments. SNR measurements demonstrated that the miniaturization process had a negligible effect (i.e., ∼1  dB losses) on the performance of the probes (Fig. 5). We used a moderate sensitivity OCT system (85 dB) for these comparative measurements; the use of a high-sensitivity OCT machine (>100  dB) could increase the precision in determining losses.

**Fig. 6:**
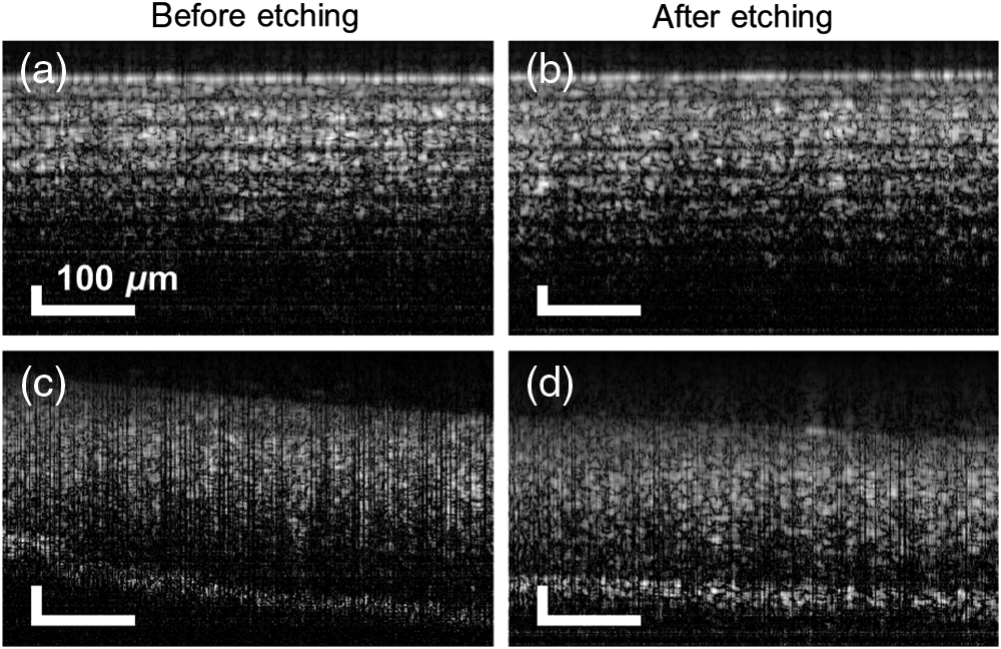
Representative B-scans of a Scotch tape roll and porcine retina acquired with a forward imaging probe [(a) and (c)] before and [(b) and (d)] after etching-enabled miniaturization.

We also performed comparative B-scan imaging of a Scotch tape roll and tissue using the probes prior and after miniaturization. Given that intraoperative guidance of intralocular tools (e.g., subretinal injection cannulas) is a promising field of applications of miniaturized probes,^14^^–^^16^ we chose the porcine retina as a tissue validation sample. The probe-sample distance was kept at 300  μm for those measurements and no averaging was applied. The reader may refer to Sec. 2 for B-scan measurements details. In accordance with the quantitative A-scan results, the acquired B-scans indicate untraceable differences in the sensitivity of the probes (Fig. 6). Both porcine retina B-scans reveal a hyper-reflective zone at the back of the retina that can be linked to the ellipsoid zones of cones and rods and the interdigitation zone/retinal pigment epithelium complex.^17^ Nevertheless, individual layers cannot be clearly identified within the hyper-reflective zone. The use of the miniaturized probes with application-tailored OCT setups/machines as well as the use of averaging and signal processing strategies can facilitate applications demanding high imaging resolution.

### 3.4 Fabrication and Performance Considerations for Miniaturized Probes

Overall, the miniaturization technique presented in this work requires two additional steps compared to the well-established technique of preparing 125-μm GRIN-based fiber probes. The first step consists in splicing and cleaving the protective CL fiber component. The second step consists of immersing the probe in an HF solution. There is a negligible cost for the materials used in these steps. However, there is a need for some additional equipment, such as an HF-resistant container, protective gloves and glasses, and a chemical fume hood. We estimate the total cost for a 10 cm probe to 4 USD. The additional fabrication turnaround time is ∼30  min (∼5  min for the splicing/cleaving steps and ∼25  min for the etching step) and can be significantly reduced by etching multiple probes at once. There is virtually no fiber length limitation for the miniaturization process, assuming that both the probe and spliced SM fiber fit in the HF-resistant container. Given that ultrathin (<125  μm) fiber handling is challenging, miniaturization of the exact required length is recommended. Note that we used inexpensive (∼1100 USD) telecom splicing/cleaving equipment in this study, which might have compromised the quality of the probes’ splicing points. The use of specialized fiber processing research workstations is recommended as it can minimize sensitivity losses. Furthermore, given that we opted for a common path configuration, there was a cost in sensitivity due to relatively low reference power. Indicative probe reference power values as a percentage of the output power ranged from 0.023% (probe 1) to 0.043% (probe used in Fig. 6) in air and from 0.00092% (probe 1) to 0.0022% (probe used in Fig. 6) for probes immersed in water. The implementation of a reference arm and/or common path technique with fixed reference power^14^ can further increase the sensitivity performance of the miniaturized probes.

## 4 Conclusion

In conclusion, we fabricated and characterized extremely miniaturized focusing GRIN-based OCT probes having approximately half the diameter of previously reported probes based on the same focusing principle (i.e., use of GRIN fiber). The developed miniaturization process is simple and controllable and does not compromise their optical performance. Similar to their thicker counterparts, the miniaturized probes have tunable and highly predictable focusing capabilities. Due to their small size, they can be encased within 41G cannulas, the smallest devices used in clinical practice as for today for minimally invasive injections. Ophthalmic, brain, and gastrointestinal tract system endoscopic tools can be benefited by this approach as the use of miniaturized probes can significantly reduce their footprint and invasiveness.
